# Black Rice Anthocyanins Suppress Metastasis of Breast Cancer Cells by Targeting RAS/RAF/MAPK Pathway

**DOI:** 10.1155/2015/414250

**Published:** 2015-11-16

**Authors:** Xiang-Yan Chen, Jie Zhou, Li-Ping Luo, Bin Han, Fei Li, Jing-Yao Chen, Yan-Feng Zhu, Wei Chen, Xiao-Ping Yu

**Affiliations:** Department of Public Health, Chengdu Medical College, Chengdu 610500, China

## Abstract

Overexpression of human epidermal growth factor receptor 2 (HER2) drives the biology of 30% of breast cancer cases. As a transducer of HER2 signaling, RAS/RAF/MAPK pathway plays a pivotal role in the development of breast cancer. In this study, we examined the molecular mechanisms underlying the chemopreventive effects of black rice anthocyanins (BRACs) extract and identified their molecular targets in HER2^+^ breast cancer cells. Treatment of MDA-MB-453 cells (HER2^+^) with BRACs inhibited cell migration and invasion, suppressed the activation of mitogen-activated protein kinase kinase kinase (RAF), mitogen-activated protein kinase kinase (MEK), and c-Jun N-terminal kinase (JNK), and downregulated the secretion of matrix metalloproteinase 2 (MMP2) and MMP9. BRACs also weakened the interactions of HER2 with RAF, MEK, and JNK proteins, respectively, and decreased the mRNA expression of* raf*,* mek*, and* jnk*. Further, we found combined treatment with BRACs and RAF, MEK, or JNK inhibitors could enhance the antimetastatic activity, compared with that of each treatment. Transient transfection with small interfering RNAs (siRNAs) specific for* raf*,* mek*, and* jnk* inhibited their mRNA expression in MDA-MB-453 cells. Moreover, cotreatment with BRACs and siRNA induces a more remarkable inhibitory effect than that by either substance alone. In summary, our study suggested that BRACs suppress metastasis in breast cancer cells by targeting the RAS/RAF/MAPK pathway.

## 1. Introduction

Breast cancer has the highest incidence rate of cancers among females in China [1]. Previous studies have shown that the human epidermal growth factor receptor 2 (HER2) was amplified or overexpressed in about 20–30% of breast cancers [2]. Furthermore, an epidemiological study found that HER2-overexpressing breast cancer is associated with a particularly aggressive form of the disease and poor prognosis [3]. Progress in this field in recent years has uncovered a plethora of mechanisms leading to the downstream signaling pathways of the HER2/neu receptor, including the phosphatidylinositol 3-kinase (PI-3K)/Akt, mitogen-activated protein kinase (MAPK), and the cyclic adenosine monophosphate (cAMP)/protein kinase A (PKA) pathways [4]. Simultaneous, expression and activation of the RAS/RAF/MAPK pathway (Mitogen activated protein kinase pathway) play an important role in the development and progression of breast cancer [5].

Anthocyanins are natural phytochemicals, which are abundantly found in black rice and are bioactive dietary agents. They have received considerable attention owing to their numerous potential health benefits including interference with several processes involved in cancer development and progression [6]. In addition, our previous studies have revealed the antiangiogenic effects of black rice anthocyanins (BRACs) extract using* in vitro* and* in vivo* model systems [7]. We recently showed that BRACs suppressed HER2^+^ breast cancer lung metastasis in a mouse model, and similar antimetastasis effects were seen in HER2^+^ breast cancer MDA-MB-453 cells treated with 200 *μ*g/mL BRACs [8]. However, the molecular mechanisms underlying the antimetastatic effects of BRACs have not been explored. Therefore, the goal of the present study was to determine the effects of BRACs on cell migration and invasion in MDA-MB-453 cells and evaluate the molecular mechanisms and possible involvement of RAS/RAF/MAPK in underlying the effects of the extract.

## 2. Materials and Methods

### 2.1. Chemicals and Reagents

BRA-90 anthocyanins (BRACs) extract was purchased from New Star (Jilin, China). Antibodies against total HER2, total K-RAS, total extracellular signal-regulated kinase 1/2 (ERK1/2), total RAF1, and phosphorylated (p)-RAF1 (Ser259) were obtained from Abcam (Cambridge, UK). Antibodies against total mitogen-activated protein kinase 1 (MEK1), p-MEK1/2 (Ser218/222), p-ERK (Thr202/Tyr204), total c-Jun N-terminal kinase 1/2 (JNK1/2), p-JNK1/2 (Thr183/Tyr185), matrix metalloproteinase (MMP2), and MMP9 were obtained from Millipore (Billerica, MA, USA). Polyethylene terephthalate membrane (8 *μ*m pore size) Millicell hanging cell culture inserts were purchased from Millipore. BD Matrigel Basement Membrane Matrix was obtained from Becton and Dickinson (Franklin Lakes, NJ, USA). The RAF inhibitor (ZM336372) was purchased from Santa Cruz Biotech (Dallas, TX, USA), MEK inhibitor (U0126) was purchased from Cell Signaling Technology (Danvers, MA, USA), and JNK inhibitor (SP600125) was purchased from Tocris (Bristol, UK). The SYBR Select Master Mix (category number 4472908),* raf*,* mek*,* jnk*, and glyceraldehyde 3-phosphate dehydrogenase (*gapdh*) universal primers, Trizol Reagent, and Lipofectamine 3000 were purchased from Invitrogen (Carlsbad, CA, USA), and the cDNA Synthesis Kit was purchased from TaKaRa Bio (Otsu, Japan).

### 2.2. Cell Culture

The human breast cancer cell lines MCF-10A and MCF-7 (HER2^−^) were kindly provided by Dr. Man-Tian Mi (Third Military Medical University of China, Chong Qing, China). MDA-MB-453 (estrogen receptor [ER]^−^, HER2/neu^+^) cells were purchased from the Institute of Biochemistry and Cell Biology, Chinese Academy of Sciences (Shanghai, China). MCF-7 and MDA-MB-453 cells were separately cultured in Dulbecco's modified Eagle's medium (DMEM)/high glucose or L-15 medium containing 10% (v/v) fetal bovine serum (FBS). MCF-10A cells were cultured in DMEM/F12 medium containing 5% (v/v) horse serum in the presence of 10 *μ*g/mL insulin, 20 ng/mL epidermal growth factor (ECF), 100 ng/mL cholera toxin, and 0.5 *μ*g/mL hydrocortisone. All cells were incubated at 37°C in a humidified atmosphere with 5% carbon dioxide (CO_2_).

### 2.3. Wound Healing (Scratch) Assay

MDA-MB-453 and MCF-10A cells were grown on 35 mm dishes to 100% confluence and then scratched using sterile pipette tips to form a wound. The cells were then treated with BRACs (0 or 200 *μ*g/mL) in the presence or absence of the RAF/MAPK inhibitors (ZM336237, U0126, and SP125600) and siRNA (*raf*-siRNA,* mek*-siRNA, or* jnk*-siRNA) for 24 h. The images were recorded using a photomicroscope (Olympus, Tokyo, Japan).

### 2.4. Cell Invasion Assay

The cell invasion assay was performed using a polyethylene terephthalate membrane (8 *μ*m pore size) with Millicell hanging cell culture inserts and BD Matrigel. BRACs (200 *μ*g/mL) and RAF/MAPK pathway inhibitors were used alone or combined with treat the cells (MDA-MB-453 and MCF-10A cells loaded into the chamber). Conditioned medium containing 10% (v/v) FBS was added to each of the wells, and the cells were allowed to invade by penetrating through the membrane during a 24 h incubation at 37°C. Then, the invading cells at the bottom of the membrane insert were detached, and the invasive potential of the cells was evaluated microscopically by counting the number of invading cells.

### 2.5. Western Blot and Immunoprecipitation

For the western blotting analysis, 100 *μ*g of protein was resolved on a 10% (v/v) trisglycine polyacrylamide gel and transferred to a polyvinylidene fluoride (PVDF). The membrane was then blocked in blocking buffer containing 5% (w/v) nonfat dry milk and 1% (v/v) Tween 20 (T) in 20 mM trisbuffered saline (TBS, pH 7.6) for 1 h at 37°C, followed by incubation with the appropriate monoclonal or polyclonal primary antibody in blocking buffer for 1 h to overnight at 4°C. This was followed by 1 h of incubation with anti-mouse or anti-rabbit secondary antibodies conjugated to horseradish peroxidase (HRP, Bio-Rad, Hercules, CA, USA), several washes, and detection using chemiluminescence (enhanced chemiluminescence, ECL Kit, Millipore), and then autoradiography using ChemiDoc XRS with the Quantity One software.

For the immunoprecipitation, IgG beads (Millipore) were incubated with antibodies against HER2, MMP2, or MMP9 for 10 min at room temperature (20~25°C), followed by treatment with equal amounts of protein (about 1000 *μ*g) for 24 h. Then, the samples were washed thrice with TBS-T buffer consisting of 187.5 mM Tris-HCl (pH 6.8), 6% (w/v) sodium dodecyl sulfate (SDS), 30% (v/v) glycerol, 150 mM dithiothreitol (DTT), 0.03% (w/v) bromophenol blue, and 0.1% (v/v) Tween 20 and then boiled in loading buffer at 80°C for 10 min. Proteins were finally resolved using western blotting as described above.

### 2.6. siRNA-Mediated Silencing of* raf*,* mek*, and* jnk *Genes

Three small interfering RNAs (siRNAs) designed to knock down the expression of the murine* raf*,* mek*, and* jnk* genes and a control siRNA with a scrambled sequence that did not specifically degrade any known cellular mRNA were purchased from Life Technologies (Carlsbad, CA, USA). MDA-MB-453 cells were transfected with the siRNAs using Lipofectamine 3000 (Life Technologies). The final siRNA concentration used for the transfection was 20 nM.

### 2.7. Quantitative Real-Time Reverse Transcription-Polymerase Chain Reaction (qRT-PCR)

Gene expression was evaluated by using quantitative real-time reverse transcription-polymerase chain reaction (qRT-PCR) analysis. Total RNA (2 *μ*g) was reverse transcribed to single-stranded cDNA using a cDNA Synthesis Kit (TaKaRa Bio, Otsu, Japan) according to the manufacturer's instructions. The qRT-PCR was performed with 20 ng of retrotranscribed RNA, 1 × SYBR Select Master Mix (Invitrogen), and the predesigned primers mix in a final volume of 10 *μ*L. Amplification reactions were carried out using a CFX Connect real-time PCR system with the following thermal profile settings: an initial step of 2 min at 95°C to activate the FastStart Taq DNA polymerase and then 40 cycles at 95°C for 15 s and 55°C for 15 s. Three independent PCR amplification experiments were performed for each transcript. The fluorescence intensities were converted into threshold cycles (Ct) using the Bio-Rad CFX Manager software; the baseline and threshold values were set automatically. Relative quantification of target gene expression was performed by 2^−ΔΔCt^ method with the average Ct values of basal samples as the calibrator for each gene.

### 2.8. Statistical Analysis

Data were expressed as mean ± standard error of the mean (SEM), either Student's *t*-test or one-way analysis of variance (ANOVA) was used for statistical analysis. All analyses were performed using the statistical package for the social sciences (SPSS) 13.0, and *P* < 0.05 was considered to be statistically significant.

## 3. Results

### 3.1. BRACs Suppressed Migration and Invasion of MDA-MB-453 HER2^+^ Breast Cancer Cells

**Figure 1:**
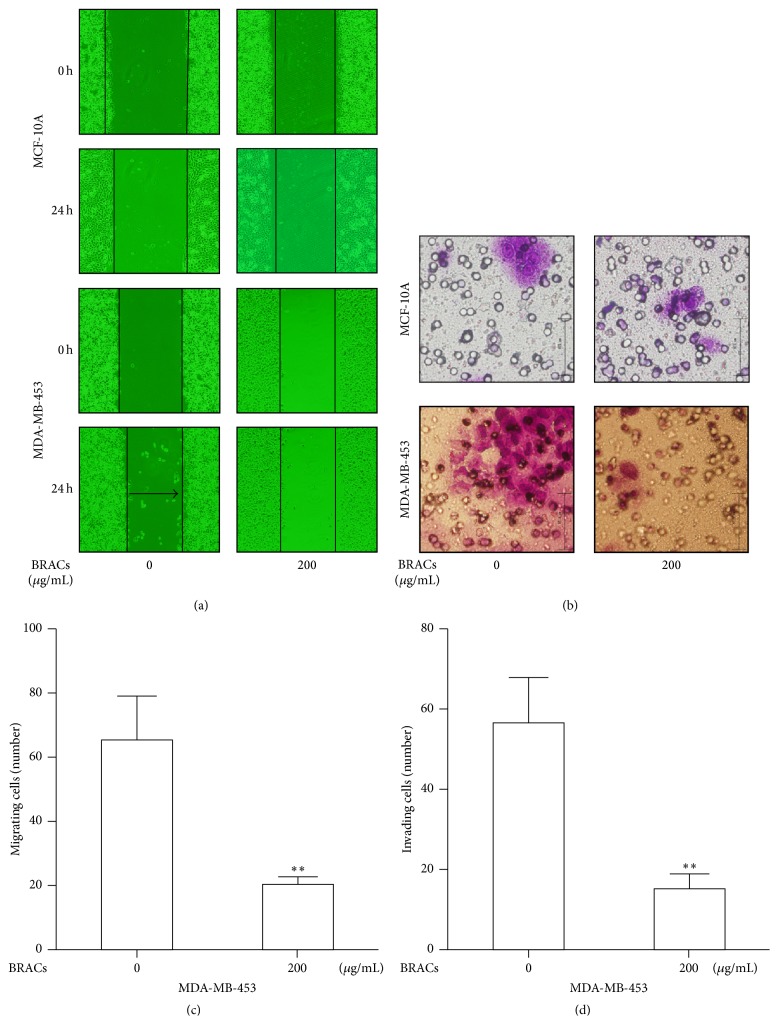
Black rice anthocyanins (BRACs) extract inhibits migration and invasion of human epidermal growth factor receptor 2 (HER2^+^) breast cancer MDA-MB-453 cell line. MCF-10A and MDA-MB-453 cells were exposed to BRACs (0 or 200 *μ*g/mL) for 24 h. (a) Cell migration was determined using wound healing migration assay (×20). (b) MCF-10A and MDA-MB-453 cells were plated in upper compartments of Matrigel invasion chambers and exposed to BRACs (0 or 200 *μ*g/mL). Then, invasive potential of treated cells was evaluated microscopically. (c) Migrating cell numbers were counted using a microscope. ^*∗∗*^ *P* = 0.005 < 0.01. (d) Number of invading cells was determined by counting using a microscope. ^*∗∗*^ *P* = 0.004 < 0.01. Data are mean ± SEM of three independent experiments.

To evaluate the potential antimetastatic effects of BRACs, we analyzed the ability to inhibit the migration and invasion of the MDA-MB-453 cell. BRACs inhibited migration and invasion of MDA-MB-453 cells while their effect against MCF-10A cells was much less potent (Figure 1).

### 3.2. BRACs Inhibited the Migration and Invasion of MDA-MB-453 through RAF/MAPK Pathway

**Figure 2:**
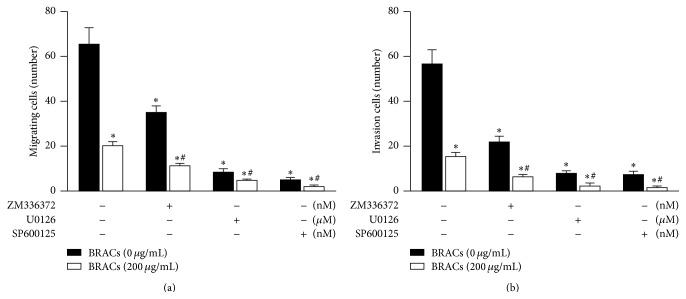
Black rice anthocyanins (BRACs) extract and rapidly RAF/MEK/JNK inhibitors decrease migration and invasion of MDA-MB-453 cells. (a) MDA-MB-453 cells were exposed to BRACs (0 or 200 *μ*g/mL) with or without RAF/MAPK inhibitors ZM33672 (70 nM), U0126 (10 *μ*M), or SP600125 (50 nM) for 24 h. Cell migration was determined using wound healing migration assay and numbers of migratory cells was determined by counting using a microscope; ^*∗*^ *P* < 0.05 versus control and ^#^ *P* < 0.05 versus BRACs groups. (b) MDA-MB-453 cells were plated in upper compartments of Matrigel invasion chambers and exposed to BRACs (0 or 200 *μ*g/mL) with or without RAF/MAPK inhibitors ZM33672 (70 nM), U0126 (10 *μ*M), or SP600125 (50 nM). ^*∗*^ *P* < 0.05 versus control; ^#^ *P* < 0.05 versusBRACs groups. Data are mean ± SEM of three independent experiments.

The addition of the RAF/MAPK inhibitors to the cell migration and invasion assay cultures reduced the migration and invasion of the MDA-MB-453 cells (Figures 2(a) and 2(b)). Furthermore, the RAF, MEK, and JNK inhibitors increased the antimetastatic effect of BRACs.

**Figure 3:**
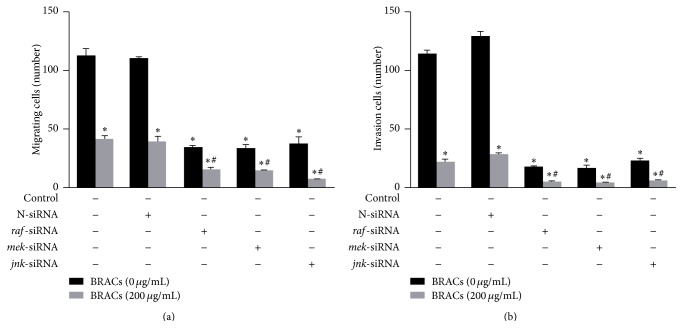
Black rice anthocyanins (BRACs) extract and small interfering RNAs (siRNA) blocked migration and invasion of MDA-MB-453 human epidermal growth receptor 2 (HER2^+^) breast cancer cells. (a) MDA-MB-453 cells were exposed to BRACs (0 or 200 *μ*g/mL) with or without transfection with* raf*-,* mek*-, or* jnk*-siRNAs for 24 h. Cell migration was determined using wound healing migration assay and number of migratory cell was counted using a microscope. ^*∗*^ *P* < 0.05 versus control and ^#^ *P* < 0.05 versus BRACs groups. (b) MDA-MB-453 cells were plated in the upper compartments of Matrigel invasion chambers and exposed to BRACs (0 or 200 *μ*g/mL) with or without transfection with* raf*-,* mek*-, or* jnk*-siRNAs. ^*∗*^ *P* < 0.05 versus control and ^#^ *P* < 0.05 versus BRACs groups. Data are mean ± SEM of three independent experiments.

In addition, transfection of MDA-MB-453 cells with* raf*-,* mek*-, and* jnk*-siRNA was performed to block the RAF/MEK/ERK pathway; we found that this decreased the invasion of MDA-MB-453 cells. Treatment with BRACs further increased the inhibitory effect of the siRNA-mediated RAF/MEK/ERK pathway blockade on the invasion of MDA-MB-453 cells (Figure 3). These results indicated that RAF, MEK, and JNK are important molecular targets of BRACs in the inhibition of cell metastasis.

### 3.3. BRACs Decreased mRNA Expression of* raf*,* mek,* and* jnk *in HER2^+^ Breast Cancer Cells

**Figure 4:**
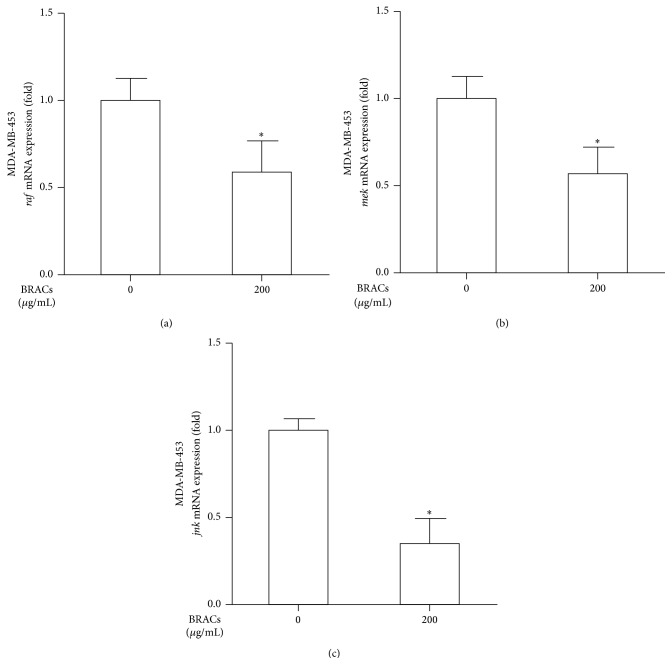
Black rice anthocyanins (BRACs) extract inhibits the mRNA expression of* raf*,* mek*, and* jnk* in MDA-MB-453 cells. MDA-MB-453 cells were treated with BRACs (0 or 200 *μ*g/mL) for 24 h. The mRNA expression of (a)* raf*, (b)* mek*, and (c)* jnk *measured using quantitative real-time reverse transcription-polymerase chain reaction (qRT-PCR). ^*∗*^ *P* < 0.05; data are mean ± SEM of three independent experiments.

**Figure 5:**
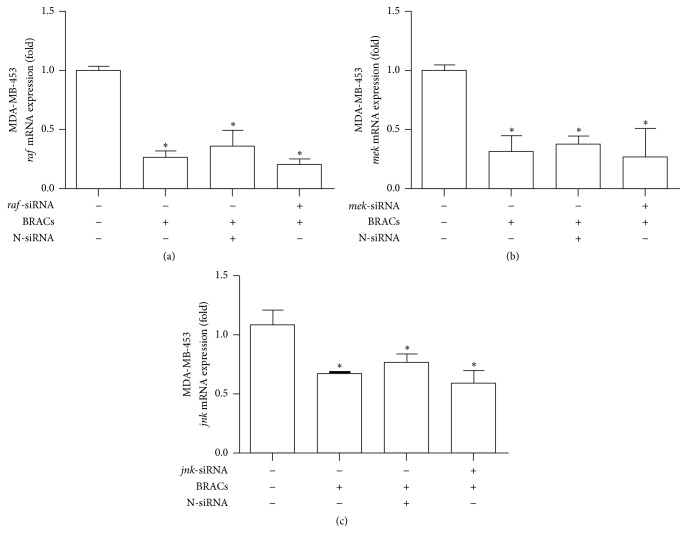
Black rice anthocyanins (BRACs) extract combined with* raf*-,* mek*-, or* jnk*-small interfering RNAs (siRNA) decreased mRNA expression of respective* genes *in MDA-MB-453 cells. MDA-MB-453 cells were treated with BRACs (0 or 200 *μ*g/mL) with or without* raf*-,* mek*-, or* jnk*-siRNAs for 24 h. mRNA expression of (a)* raf*, (b)* mek*, (c)* jnk* was measured using quantitative real-time reverse transcription-polymerase chain reaction (qRT-PCR). ^*∗*^ *P* < 0.05; data are mean ± SEM of three independent experiments.

To confirm the molecular mechanisms underlying the antimetastatic effects of BRACs, we investigated whether treatment with BRACs inhibited the mRNA expression of* raf1*,* mek*, and* jnk* in HER2^+^ breast cancer cells. As shown in Figure 4, we observed significant inhibition of* raf1*,* mek*, and* jnk *expression in siRNA-treated MDA-MB-453 cells. More interestingly, the expression of these genes was further reduced significantly when BRACs was applied in combination with the siRNAs (Figure 5).

### 3.4. BRACs Treatment Inhibited Protein Expression of K-RAS and Phosphorylation of RAF and MAPKs in HER2^+^ Breast Cancer Cells

**Figure 6:**
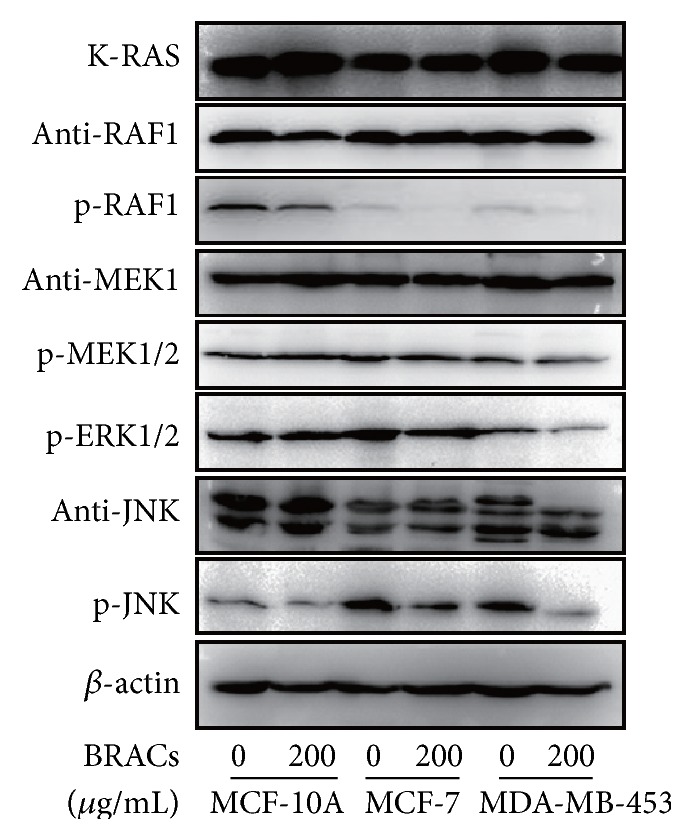
Effects of black rice anthocyanins (BRACs) extracts on phosphorylation of RAS, RAF, MEK, ERK, and JNK. MCF-10A, MCF-7, and MDA-MB-453 cells were treated with BRACs (0 or 200 *μ*g/mL) for 24 h. Phosphorylation of RAF, MEK, ERK, and JNK was analyzed by immunoblotting. Expression of *β*-actin served as an internal control.

We investigated the effects of BRACs on the kinase activity of K-RAS, an upstream kinase of RAF1. The western blot assay revealed that BRACs strongly suppressed K-RAS activity in MDA-MB-453 cells (Figure 6). BRACs also decreased the phosphorylation of MEK1/2, ERK1/2, JNK, and RAF1, which was affected most. These data indicate that BRACs inhibited the phosphorylation of upstream kinases more strongly than that of downstream kinases.

**Figure 7:**
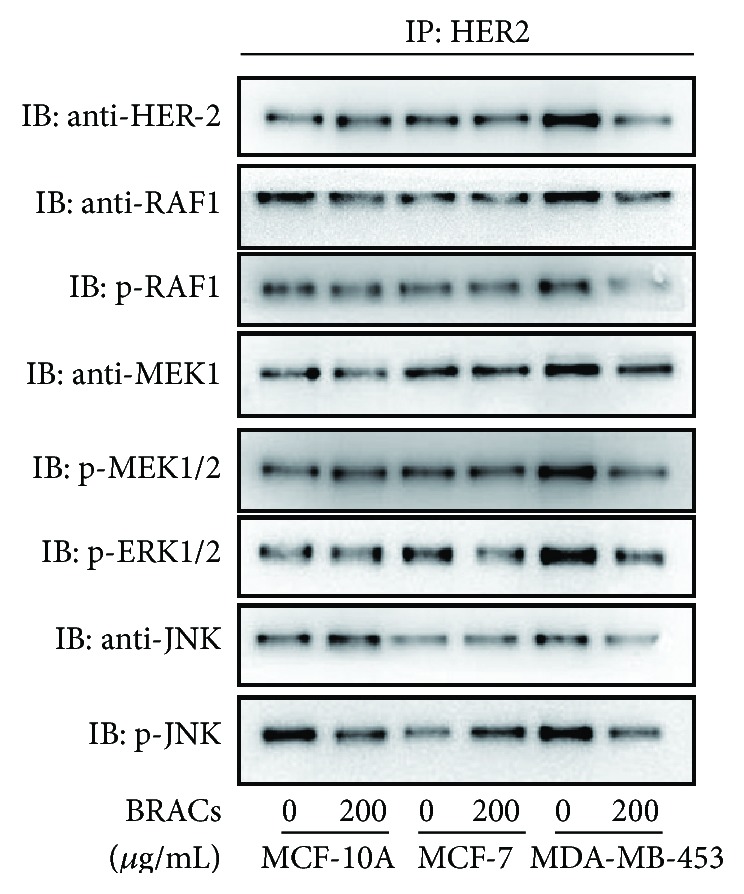
Effects of BRACs on the interactions of HER2 with RAF, MEK, ERK, and JNK. MCF-10A, MCF-7, and MDA-MB-453 cells were treated with BRACs (0 or 200 *μ*g/mL) for 24 h. Cell lysates were collected and immunoprecipitated with anti-HER2 antibody and then immunoblotted with antibodies against HER2, RAF/phosphorylated (p)-RAF, MEK/p-MEK/p-ERK, and JNK/p-JNK.

To determine whether the inhibitory effect of BRACs on RAF/MAPK signaling is due to the direct physical interaction of HER2 and RAF/MAPK proteins, we performed an* in vitro* immunoprecipitation (IP) assay. The results indicated that BRACs inhibited the interactions between HER2 and RAF1, MEK, and JNK (Figure 7). These results suggested that BRACs might bind to HER2 as well as RAF1, MEK, or JNK or all the three at allosteric sites.

### 3.5. Cotreatment with Inhibitors or siRNA and BRACs Suppressed Metastasis in HER2^+^ Breast Cancer Cells

**Figure 8:**
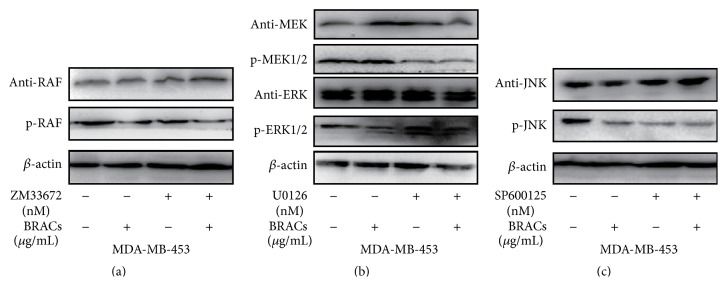
Effects of black rice anthocyanins (BRACs) extract and RAF/MAPK inhibitors on phosphorylation of RAF, MEK, ERK, and JNK. MDA-MB-453 cells were treated with BRACs (0 or 200 *μ*g/mL) with or without inhibitors ZM33672 (70 nM), U0126 (10 *μ*M), and SP600125 (50 nM) for 24 h. Phosphorylation of (a) RAF, (b) MEK1/2, ERK1/2, and (c) JNK was determined using immunoblotting. Expression of *β*-actin served as an internal control.

As shown in Figure 8, treatment with inhibitors attenuated the phosphorylation of RAF1, MEK1/2, ERK1/2, and JNK. Furthermore, RAF/MAPK pathway inhibitors suppressed the expression of the various proteins in the RAF/MAPK pathway. We subsequently evaluated the effects of BRACs and RAF/MAPK pathway inhibitors on the activation of the same proteins in MDA-MB-453 cells. Strikingly, cotreatment with BRACs and MAPK inhibitors downregulated the phosphorylation of RAF/MAPK pathway proteins with a significantly greater potency than that shown by BRACs alone in MDA-MB-453 cells.

**Figure 9:**
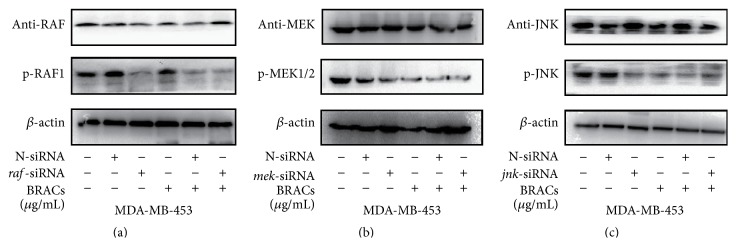
Effects of black rice anthocyanins (BRACs) extract and small interfering RNA (siRNA) on the activation of RAF/MAPK in MDA-MB-453 cells. MDA-MB-453 cells were treated with BRACs (0 or 200 *μ*g/mL) with or without* raf*-,* mek*-, or* jnk*-siRNAs for 24 h. Phosphorylation of (a) RAF, (b) MEK1, and (c) JNK was determined using immunoblotting (IB). Expression of *β*-actin served as an internal control.

In addition, we detected phosphorylation of RAF1, MEK, and JNK1/2 kinase in cells, while treatment with their respective siRNAs downregulated p-RAF, p-MEK, and p-JNK protein expression in cell lysates. Interestingly, the cotreatment decreased the phosphorylation of RAF, MEK, and JNK more significantly than using either substance alone (Figure 9).

### 3.6. BRACs Treatment Inhibited the Protein Expression of MMP2 and MMP9 in HER2^+^ Breast Cancer Cells

**Figure 10:**
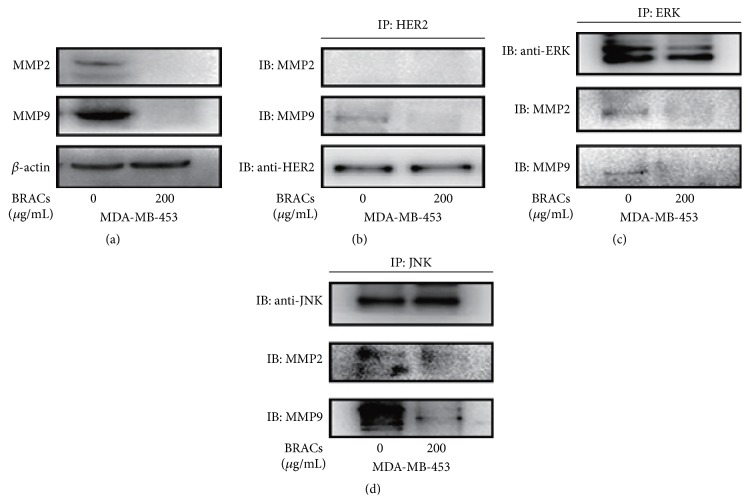
Effects of black rice anthocyanins (BRACs) extract on MMP2 and MMP9. (a) MDA-MB-453 cells were treated with BRACs (0 or 200 *μ*g/mL) for 24 h. Cells were harvested for analysis of MMP2 and MMP9 expression using immunoblotting. Expression of *β*-actin served as a loading control. (b) MDA-MB-453 cells were treated with BRACs (0 or 200 *μ*g/mL) for 24 h. Cell lysates were collected and immunoprecipitated with anti-HER2 antibody and then immunoblotted with antibodies against MMP2, MMP9, and HER2. (c) MDA-MB-453 cells were treated with BRACs (0 or 200 *μ*g/mL) for 24 h. Cell lysates were collected and immunoprecipitated with an anti-ERK antibody and then immunoblotted with antibodies against MMP2, MMP9, and ERK. (d) MDA-MB-453 cells were treated with BRACs (0 or 200 *μ*g/mL) for 24 h. Cell lysates were collected and immunoprecipitated with an anti-JNK antibody and then immunoblotted with antibodies against MMP2, MMP9, and JNK.

To confirm that BRACs inhibit metastatic signaling, we examined their potential inhibition of MMP2 and MMP9. Treatment of MDA-MB-453 cells with BRACs completely blocked the expression of MMP2 and MMP9 proteins (Figure 10).

Next, to examine the mechanism by which BRACs inhibited the interactions of MMP2 and MMP9 with HER2, ERK, and JNK, we pretreated MDA-MB-453 cells with or without BRACs for 24 h and then performed an immunoprecipitated assay with MMP2 and MMP9 antibodies followed by analysis using immunoblotting. We observed that BRACs inhibited the interaction of MMP2 and MMP9 with HER2, ERK, and JNK (Figures 10(b)–10(d)). In particular, BRACs markedly inhibited the interaction between MMP9 and JNK (Figure 10(d)).

## 4. Discussion

Anthocyanins are a class of flavonoids, which include reddish natural pigments that are extensively distributed in fruits and flowers. The BRACs contain anthocyanins such as cyanins, peonidins, cyanidin-3-glucose, and peonidin-3-glucose. Epidemiological studies have positively correlated the dietary consumption of anthocyanins with reduced cardiovascular disease-associated mortality [9] and certain types of cancer [6, 7, 10]. Previous findings have suggested the potential of anthocyanins or anthocyanin-derived pigments for use in the chemotherapy of breast cancer [6–10].

Metastatic dissemination is an extremely complex and highly organized and organ-specific process that involves numerous reciprocal interactions between cancer cells and the normal cells of the host. Breast cancer progression is dependent on the capacity of cancer cells to metastasize to distant organs; dysregulation of gene expression associated with the RAS/RAF/MAPK pathway plays a key role in this process [11]. Importantly, HER2/Neu overexpression is closely linked to the dysregulation of this pathway. In this study, we determined whether BRACs inhibited breast cancer cell invasion by suppressing RAS/RAF/MAPK signaling, and the possible involvement of MMP2 and MMP9, which are regulated by JNK, was involved in the antimetastatic activity of BRACs.

Previous reports have suggested that K-RAS kinase plays an important role in regulating HER2^+^ breast cancer cell metastasis [12]. We previously found that BRACs downregulated the expression of K-RAS in MDA-MB-453 cells. Furthermore, RAF kinases play a central role in the RAS/RAF/MEK/JNK signaling pathway, and RAF is activated by guanosine triphosphate- (GTP-) bound RAS [13]. Following activation, RAF phosphorylates MEK, which on activation subsequently phosphorylates and activates MAPK, which has multiple targets and leads to changes in gene transcription, resisting apoptosis, and enhancing metastasis [14]. In our study, we showed that oncogenic signaling by RAF1 is linked to the activation of the MEK/JNK pathway in metastatic breast cancer cells. In these experiments, BRACs were shown to decrease invasiveness, phosphorylation of RAF1, and* raf1* mRNA expression in MDA-MB-453 cells. Furthermore, cotreatment with BRACs and an RAF inhibitor or* raf*-siRNA induced a stronger inhibitory effect than that obtained with any of these substances alone. In agreement with our results, Jong-Eun Kim has also implicated the RAF1 pathway in the MAPK signaling in skin cancer cells and found that cyanidin suppresses ultraviolet B-induced skin cancer by targeting RAF1 [15]. Our findings are also consistent with, but not limited to, this result. New discoveries from our previous research showed that BRACs act synergistically with the RAF inhibitor and siRNA to further inhibit invasiveness and potentiate the antitumorigenic activity against HER2-overexpressing breast cancer cells.

MEK1 is a tyrosine (Y-) and S/T-dual specificity protein kinase [16], and its activity is positively regulated by RAF phosphorylation on serine residues in the catalytic domain. Our data also showed that BRACs inhibited the phosphorylation of MEK1 and* mek1* mRNA expression. Furthermore, cotreatment with BRACs and inhibitors or siRNAs exhibited stronger antimetastatic effects in MDA-MB-453 cells than any treatment alone did. These findings are correlated with a reduced pulmonary metastatic burden. Moreover, the predominant downstream target of MEK1 is ERK, and BRACs treatment decreased the phosphorylation of ERK1/2.

In individual tumor types, JNK may not play a role in tumor development or may contribute (positively or negatively) to tumor pathology. Previous studies have established that JNK signaling is required for normal mammary gland development and that it has a suppressive role in mammary tumorigenesis [17].

In contrast, we discovered that JNK promoted the invasion of breast cancer cells. Our findings are also consistent with recent discoveries of Cellurale et al. [18], which demonstrated that JNK plays a key role in K-RAS-induced tumorigenesis. These results may explain the association between K-RAS mutations and HER2^+^ breast cancer. We reported that BRACs suppressed the phosphorylation of JNK and* jnk* mRNA expression. Similarly, SP600125 (JNK inhibitor) or* jnk*-siRNA downregulated JNK phosphorylation and* jnk* mRNA expression in both the presence and the absence of BRACs.

HER2 is a well-known upstream kinase of RAF/MAPK, and, therefore, we examined whether BRACs affected the interaction of HER2 with RAF1, MEK1, ERK1/2, and JNK. We found that BRACs inhibited the binding between each of these kinases with HER2. Therefore, we concluded that the BRACs-mediated inhibition of interactions between HER2 and RAF, MEK, ERK, and JNK subsequently inhibited the metastasis of MDA-MB-453 cells.

MMP2 and MMP9 play vital roles in tumor metastasis and invasion via the degradation of various proteins of the extracellular matrix and destruction of histological barriers [19]. Our study investigated whether HER2^+^ breast cancer cell metastasis was affected by the RAF/MAPK signaling pathway targeted by BRACs treatment. We found that treatment with BRACs decreased the expression of MMP2 and MMP9. Furthermore, the immunoprecipitation assay revealed interactions between each of the HER2, ERK, and JNK proteins and MMP9, which is in agreement with the results of Cho et al. [20]. This finding suggests that HER2 can regulates the expression of MMP9 via the RAF/MAPK signaling pathway. Moreover, BRACs treatment inhibited the HER2/MAPK/MMP9 signaling pathway, leading to the suppression of metastasis in HER2^+^ breast cancer cells. The ERK signaling pathway is known to upregulate the expression of MMPs [21]. However, we found that JNK showed the highest level of binding to MMP2 and MMP9 among the proteins we investigated.

Collectively, these results demonstrate the potential antimetastatic effect of BRACs treatment mediated via RAS/RAF/MAPK signaling in HER2^+^ breast cancer cells. In addition, BRACs treatment inhibited the activation and mRNA expression of key components of the RAF/MAPK signaling pathway. Furthermore, it decreased the interactions of HER2 with downstream signaling components as well as those of MMP2 and MMP9 with their upstream regulators.
